# Severe tinnitus in a patient with acquired deafness for over 50 years: a case report

**DOI:** 10.1186/s13030-018-0136-x

**Published:** 2018-11-26

**Authors:** Saeko Matsuzaki, Naoki Oishi, Kaoru Ogawa

**Affiliations:** 1 Department of Otolaryngology, Sanno Hospital, Tokyo, Japan; 2 Department of Otolaryngology, Head and Neck Surgery, Keio University School of Medicine, 35 Shinamomachi, Shinjuku, Tokyo, 1608582 Japan

**Keywords:** Tinnitus, Deaf, Palpitation, Antidepressant, Autogenic training

## Abstract

**Background:**

There have been many reports on the treatment effect of cochlear implantation and hearing aids in the treatment of tinnitus in patients with severe hearing loss. However, as far as we are aware, there are no reports of investigation of treatment approaches for the tinnitus of deaf patients whose communication is solely carried out in sign language due to a long duration of deafness.

**Case presentation:**

We experienced a case of severe tinnitus with bilateral deafness for more than 50 years. The patient is a 69-year-old woman who communicates with her family solely in sign language. Family stress triggered the onset of tinnitus, accompanied by sleep disorder and palpitations. At the initial visit, she suffered from severe tinnitus (THI 94) as well as strong tendencies toward depression and anxiety. Because neither the patient nor her family was willing to use cochlear implantation, the administration of an antidepressant and a sleep-inducing agent was started, which resulted in improvement of the psychological conditions. Tinnitus distress, synchronized with the heartbeat, was relieved by the addition of autogenic training. At four and half years after the initial visit, the THI score had dropped to 0, and the subjective tinnitus and palpitation had almost disappeared, with only a low dose of antidepressant necessary.

**Conclusion:**

A deaf patient with severe tinnitus was successfully treated with drug and psychotherapy.

## Background

There have been many reports on the treatment effect of cochlear implantation on tinnitus in patients with the severe hearing loss [1–5]. For hearing loss cases in which the hearing of the patients can be aided by a hearing aid, there are several reports that investigated the treatment effect of wearing a hearing aid on tinnitus [6–8]. However, as far as we are aware, there are no reports that have investigated treatment approaches for the tinnitus of deaf patients whose communication is solely carried out in sign language due to a long duration of deafness.

Here we present a case of a patient with severe tinnitus who has presumably been deaf for more than 50 years. The patient communicates with her family and those around her solely in sign language, and neither she nor her family was willing to have a cochlear implantation done. Here we report a rare case of a patient with bilateral deafness in which treatment approaches other than cochlear implantation were effective against tinnitus.

## Case presentation

The patient is a 69-year-old woman. She was infected with pneumonia at the age of three and had a high fever for a long period. After the fever abated, she became aware of hearing loss. Because she lived in a mountain village, she rarely visited medical facilities. Since then, she had never worn a hearing aid, had gone to a school for the deaf from elementary school to high school, and communicated with people in sign language. Her husband also used sign language, so she had not used oral communication from elementary school to the present. In 2011, family stress triggered the onset of tinnitus. Because around the same time, she began suffering from sleep onset disorder (it took about one hour until sleep onset), nocturnal awakening, and palpitations, she went to a nearby psychosomatic medicine clinic. She received medication at the clinic but her tinnitus did not improve, so she was referred to our department in 2014.

Various questionnaires were given at her consultation. The result of THI (Tinnitus Handicap Inventory) [9], which is used to evaluate the severity of tinnitus, was a high of 94, which indicates the most severe form of tinnitus. Also, an SDS (Self-rating Depression Scale) [10] score, which is used to measure depression tendency, was as high as 61, showing that she had a tendency toward depression. The STAI (State-Trait Anxiety Inventory) [11], which is used to measure anxiety tendency, returned a State Anxiety (STAIs) score of 69 and a Trait Anxiety (STAIt) score of 67, indicating that she had an extremely high anxiety tendency. As one of the personal features of her tinnitus, she had no concept of the “loudness of tinnitus” because she had been deaf since childhood.

In imaging tests, there were no abnormal findings except for a slight enlargement of the inner ear canals observed by temporal bone CT scan. Head MRI showed no abnormal findings within the skull or in the internal auditory canals.

For treatment, we started oral administration of a serotonin reuptake inhibitor (SSRI) (paroxetine hydrochloride, Paxil®, 12.5 mg, started as one tablet a day, increased to three tablets a day) and a sleep-inducing agent (suvorexant, Belsomra®, 15 mg, one tablet a day). One and a half months later, the sleep onset disorder and nocturnal awakening improved, but early morning arousal persisted. Then, a benzodiazepine anxiolytic was added (etizolam, Depas®, 0.5 mg, one tablet a day). At four months after the initial visit, THI was 84, SDS 43, STAIs 50, and STAIt 48, which showed that her psychological condition had improved, although the tinnitus distress level did not change.

At this time, there was no improvement in perceived palpitations, and “pulsatile tinnitus” that seemed to synchronize with the heartbeat became the chief complaint concerning tinnitus, which led us to suspect that she had autonomic disorders. Six months after the initial visit, she started to receive psychotherapy (autogenic training). After the start of the treatment, we treated the patient with psychotherapy once a month, which continued until the 7th therapy session was completed. At the end of psychotherapy, our test results showed THI at 60, SDS 45, STAIs 32, and STAIt 43, showing a further improvement trend. The THI score was still high at 60, but the subjective tinnitus distress became “not so annoying,” and the “echoing tinnitus” that was the cause of the patient’s discomfort at the time of the initial visit disappeared. Only the pulsatile tinnitus, which seemed to be related to palpitations, remained.

One year and seven months after the initial visit*,* palpitations and pulsatile tinnitus, as well as anxiety and insomnia, were aggravated due to work stress. She restarted psychotherapy. At the same time, SSRIs were replaced by noradrenergic and specific serotonergic antidepressants (NaSSAs) (mirtazapine, Reflex®, 15 mg, started as one tablet a day, increased to two tablets a day). As a result, improvement of the palpitations and insomnia gradually occurred, and two years and one month after the initial visit, our test results showed THI at 40, SDS 47, STAIs 40, and STAIt 46.

By three years after the initial visit, the symptoms had stabilized and the anxiolytic drug was discontinued, but oral administration of the NaSSA and sleep induction drugs continued. The subjective tinnitus and palpitations at the time of sleep almost disappeared, and the sleep onset disorder and nocturnal awakening rarely occurred.

Now 4.5 years have passed and she is taking only a low dose NaSSA (mirtazapine, Reflex®, 15 mg, 0.5 Tablets a day). The latest test results were THI 0, SDS 43, STAIt 47, and STAIs 50, indicating that the tinnitus distress had disappeared completely.

## Discussion

It has been reported that many people with hearing loss are aware of tinnitus [12]. Although there have been some reports that investigated the effects of hearing aids and cochlear implantation in cases in which tinnitus interferes with patients’ daily life activities [1, 4, 5], no reports that examined tinnitus treatments for deaf people who do not select cochlear implantation as a treatment choice have been published. As for the mechanism by which tinnitus occurs, the neurophysiological model proposed by Jastreboff in the 1980’s is widely known. In this model, it is suggested that tinnitus development involves not only the auditory pathway but also other structures such as the limbic system [2, 13–15]. This model explains the reason increased depression and anxiety aggravate discomfort caused by tinnitus [8].

In this case, the patient had been deaf since childhood, and family stress triggered the onset of tinnitus with strong discomfort. Psychologically, she seemed to have strong tendencies toward depression and anxiety, which may have increased the discomfort of tinnitus. It is presumed that she has been deaf for more than 50 years, and all communication with her family members and those around her is done by sign language. These background factors made the therapeutic effect of cochlear implantation questionable. Also, neither the patient nor her family was willing to have a cochlear implantation done only for the treatment of tinnitus. Accordingly, the patient started drug and psychotherapy to treat psychological and autonomic disorders, such as anxiety, depression, sleep onset disorder, and palpitations. Improvement was seen in the scores of various questionnaires as soon as one month after the beginning of treatment, and a marked improvement was observed one year later (Table 1). After that, drug adjustment and the addition of psychotherapy at the time of repeated deterioration of symptoms caused by additional stress resulted in improvement. Now, four and half years after the initial visit, the tinnitus distress has completely disappeared.

**Table 1:** THI, SDS and STAI score progression

	at first visit	at 4 months	at 1 year	at 2 years	at 4 years	at 4.5 years
THI score	94	84	60	40	14	0
SDS score	61	43	45	47	42	43
STAIs score	69	50	32	40	36	47
STAIt score	67	48	43	46	32	50

Among deaf people, the prevalence of depression and other psychiatric diseases is high, and one study reported that the lifetime rate of attempted suicide was 30% [16]. The tinnitus of deaf people is thought to be strongly related to their psychological state, such as anxiety, depression, and insomnia. As previously reported, behavior therapy training, autogenic training, and structured group psychotherapy show only short-term success [17]. However, our case indicates the long-term clinical usefulness of a combination of psychotherapy and medication to treat the tinnitus of deaf patients for whom cochlear implantation is not a desirable choice.

## Conclusions

As seen in this case, for a deaf patient who has been deaf for such a long period of time that cochlear implantation is not a desirable choice of treatment, drug therapy such as antidepressant drugs and psychotherapy focusing on the patient’s mental state can be effective in the treatment of tinnitus. Further study including more cases will be needed to further clarify the mechanisms of the tinnitus of deaf patients and to provide even more effective treatments.

## Abbreviations

NaSSAs: Noradrenergic and Specific Serotonergic Antidepressants
SDS: Self-rating Depression Scale
SSRIs: Selective Serotonin Reuptake Inhibitors
STAI: State-Trait Anxiety Inventory
STAIs: STAI-state
STAIt: STAI-trait
THI: Tinnitus Handicap Inventory
